# The Effect of Surface Treatment with Isocyanate and Aromatic Carbodiimide of Thermally Expanded Vermiculite Used as a Functional Filler for Polylactide-Based Composites

**DOI:** 10.3390/polym13060890

**Published:** 2021-03-14

**Authors:** Mateusz Barczewski, Olga Mysiukiewicz, Aleksander Hejna, Radosław Biskup, Joanna Szulc, Sławomir Michałowski, Adam Piasecki, Arkadiusz Kloziński

**Affiliations:** 1 Institute of Materials Technology, Poznan University of Technology, Piotrowo 3, 61-138 Poznan, Poland; olga.mysiukiewicz@put.poznan.pl (O.M.); radek.biskup@op.pl (R.B.); 2 Department of Polymer Technology, Gdańsk University of Technology, Narutowicza 11/12, 80-233 Gdańsk, Poland; aleksander.hejna@pg.gda.pl; 3 Faculty of Chemical Technology and Engineering, UTP University of Science and Technology, Seminaryjna 3, 85-326 Bydgoszcz, Poland; Joanna.szulc@utp.edu.pl; 4 Department of Chemistry and Technology of Polymers, Cracow University of Technology, Warszawska 24, 31-155, Kraków, Poland; spri@chemia.pk.edu.pl; 5 Institute of Materials Engineering, Poznan University of Technology, Jana Pawła II 24, 60-965 Poznan, Poland; adam.piasecki@put.poznan.pl; 6 Institute of Chemical Technology and Engineering, Poznan University of Technology, Berdychowo 4, 60-965 Poznan, Poland; arkadiusz.klozinski@put.poznan.pl

**Keywords:** polylactide, vermiculite, composite, surface treatment, mechanical properties

## Abstract

In this work, thermally expanded vermiculite (TE-VMT) was surface modified and used as a filler for composites with a polylactide (PLA) matrix. Modification of vermiculite was realized by simultaneous ball milling with the presence of two PLA chain extenders, aromatic carbodiimide (KI), and 4,4’-methylenebis(phenyl isocyanate) (MDI). In addition to analyzing the particle size of the filler subjected to processing, the efficiency of mechanochemical modification was evaluated by Fourier transform infrared spectroscopy (FTIR) and scanning electron microscopy (SEM). The composites of PLA with three vermiculite types were prepared by melt mixing and subjected to mechanical, thermomechanical, thermal, and structural evaluation. The structure of composites containing a constant amount of the filler (20 wt%) was assessed using FTIR spectroscopy and SEM analysis supplemented by evaluating the final injection-molded samples’ physicochemical properties. Mechanical behavior of the composites was assessed by static tensile test and impact strength hardness measurements. Heat deflection temperature (HDT) test and dynamic thermomechanical analysis (DMTA) were applied to evaluate the influence of the filler addition and its functionalization on thermomechanical properties of PLA-based composites. Thermal properties were assessed by differential scanning calorimetry (DSC), pyrolysis combustion flow calorimetry (PCFC), and thermogravimetric analysis (TGA). The use of filler-reactive chain extenders (CE) made it possible to change the vermiculite structure and obtain an improvement in interfacial adhesion and more favorable filler dispersions in the matrix. This translated into an improvement in impact strength and an increase in thermo-mechanical stability and heat release capacity of composites containing modified vermiculites.

## 1. Introduction

Polylactide (PLA) is a biobased polymer that attracted attention recently because of its good performance and processing properties, and relatively high availability compared to the rest of the commercially used biodegradable thermoplastic polyesters [1,2]. Despite many advantages that made PLA an exceedingly popular polymer, it also has some disadvantages that significantly limit its use. These include susceptibility to hydrolytic degradation during melt processing, high brittleness, and low glass transition temperature [2,3,4]. For two decades, scientists have been developing PLA modification methods that allow for the adjustment of its properties to increase the range of applicability. The most common modifications to the structure of the polylactide itself can be carried out by heterogeneous nucleation realized in order to increase crystallinity [5,6] or the use of chain extenders to increase its molecular weight [7,8,9,10]. Another solution that allows increasing the range of applicability, mostly from the point of view of toughness improvement, is the production of reactive mixtures of polylactide with other biodegradable polymers [11,12]. The production of PLA-based composites using inorganic fillers created many possibilities for modifications, resulting in a significant range of applicability of this polymer [13]. The addition of fillers makes it possible to modify the crystalline structure; improve thermomechanical properties [14,15,16], thermal conductivity [17], and barrier properties [18]; as well as decrease flammability [19]. Moreover, in the case of using fibrous fillers, such as glass or basalt fibers, a significant improvement of the mechanical properties is noted [20,21,22]. Unfortunately, due to inorganic fillers’ hydrophilic nature, both powdery and fibrous, there can be an insufficient adhesion at the polymer–filler interface and the possible presence of moisture in the filler or the polymer. The presence of even the lowest moisture content during the technological process carried out in the molten state usually leads to the phenomenon of hydrolytic degradation, resulting in chain scission of all polymers from the group of thermoplastic polyesters, including PLA [19,23]. Therefore, one of the more developed issues related to polymer composites’ production is the modification of the surface of fillers and development of methods to reduce their hydrophilicity [24,25,26,27].

Vermiculite (VMT) is a clay material from the 2:1 group of phyllosilicates, characterized by Al octahedral sheets, placed between two Si tetrahedral sheets, which together create a single layer of the VMT [28]. Compared to other commonly used layered silicates such as montmorillonite, saponite, or hectorite, vermiculite is much cheaper. It has a much larger cation exchange capacity (CEC), which explains the more difficult exchange of cations and organic molecules due to higher interlayer charge density [28,29]. Moreover, vermiculite, unlike other phyllosilicate clays, can be thermally expanded, just like expanded graphite. Hence, it is possible to use it as an excellent insulator with high thermal stability. In addition, the possibility of exfoliation makes it a filler that can be used to create various composite and nanocomposite materials with novel functionalities [30,31].

In most cases, surface modification of inorganic fillers allows for the reduction of their hydrophilicity or surface functionalization, which directly increases its compatibility with selected groups of polymers. The most effective methods of surface modification include silanization processes [32,33], stearic acid treatment [34,35], the use of diazonium salts [36,37], and the use of isocyanates [38,39]. To use the potential resulting from the VMT structure, various modification methods leading to the exfoliation and intercalation of the layered filler structure have been used. Fernández and Fernández [28] introduced organomodified vermiculite (OVMT) to improve the degradation of polylactide in alkaline medium. The OVMT used in their study was modified using Oleylbis(2-hydroxyethyl)methylammonium chloride (ETO), octadecyltrimethylammonium bromide (ODTMA), and polyhedral oligomeric silsesquioxane mono-substituted with an aminopropylheptaisooctyl-functional group. In another work, Fernández et al. [40] focused on properties of PLLA nanocomposites manufactured by the incorporation of OVMT modified with (γ-glycidoxypropyl) trimethoxy silane (GPS), in the presence of hexadecyltrimethyl ammonium bromide (HDTMA). The low amounts of the intercalated and grafted filler allowed the improvement of thermal stability of the biodegradable polymer and the achievement of nucleating effects on PLLA. Guo and co-workers studied the effect of vermiculite incorporation into PLA-based composites manufactured by solvent processing on its biodegradability [41]. The results showed that along with the addition of organomodified vermiculite, the biodegradability of nanocomposites was improved, mainly due to the lower degree of the crystallinity of the modified materials in comparison to unmodified PLA. The crystalline structure of PLA modified with ammonium acetate organic intercalated vermiculite used as a nucleating agent was evaluated by Li et al. [42]. The filler prepared by solution intercalation and amidation reaction with ammonium acetate makes it possible to restrict the PLA cold crystallization and improve the crystallinity by more than 37%. To summarize, most of the described works that focus on the modification of vermiculite are aimed at its intercalation with appropriate chemical treatment. In the literature, however, there are relatively few works focused on thermally expanded vermiculite (TE-VMT) and methods of its modification in terms of applications as a cost-effective filler for thermoplastic composites.

According to Li et al., the application of ball milling procedure reduces twice the surface area and the porosity of expanded vermiculite [43]. Considering the development of the polymeric composites by melt processing, this effect may be beneficial from the point of view of improved adhesion with the polymer and reduced moisture absorption. Wang et al., in their work [44], compare the hot solution method with ball milling processing of VMT to obtain OVMT. Their investigations suggest that the application of the novel ball milling method allows for a large interlayer space in OVMT and much higher residual energy of the filler, which, according to the authors, results in high sensitivity for the absorption of other molecules.

Based on the possibilities of simultaneous changes in the structure of vermiculite and its surface modification described in the literature, we decided to try to use ball milling with two commonly used chain extenders, which are reactive simultaneously to the polymer and the filler, in order to increase the compatibility between PLA and TE-VMT. The literature describes the possibilities of using isocyanates as chain extenders and agents enabling the modification of fillers’ surface; among them, the isocyanates used and the commercial compound under the name BioAdimide^®^ have attracted particular attention. As part of this research work, we assumed that the high reactivity of isocyanate groups to the hydroxyl groups contained in fillers would allow for obtaining interlayers with active NCO- groups on the TE-VMT surface. Liu et al. presented in their studies the application of hexamethylene diisocyanate (HDI) as an effective chain extender for low molecular weight PLA [45]. Hao et al. discussed another example of using polyaryl polymethylene isocyanate (PAPI) as a chain extender for PLA [7]. Their results showed the improvement of the polymer’s molecular weight after modification, evaluated by molten state rheological experiments. Laske et al. used the commercial additive BioAdimide^®^ 500XT as a chain extender for PLA, which shows the limited effect on the polymeric matrix structure [46]. Besides that, Awal and co-workers used BioAdimide^®^ 500 as an additive for PLA–wood fiber composite, which improves the processability and interfacial adhesion [8], which allows BioAdimide^®^ to be determined as a multipurpose modifier that will be suitable for modification of inorganic filler in the mechanochemical synthesis process.

This work is aimed to evaluate the possibility of simultaneous ball milling of expanded vermiculite with chain extenders to manufacture the filler for PLA-based composites. The influence of the one-step milling/modification process on the compatibility of the filler with polymeric matrix and composites’ structural changes resulting in mechanical, thermal, and thermomechanical properties was investigated.

## 2. Experimental

### 2.1. Materials and Sample Preparation

Ingeo^TM^ 3100HP polylactide (Nature Works, MN, USA) with a mass flow rate (MFR) of 24 g/10 min (2.16, 210 °C) and density of 1.24 g/cm^3,^ was used as the matrix polymer of the composites.

**Figure 1:**
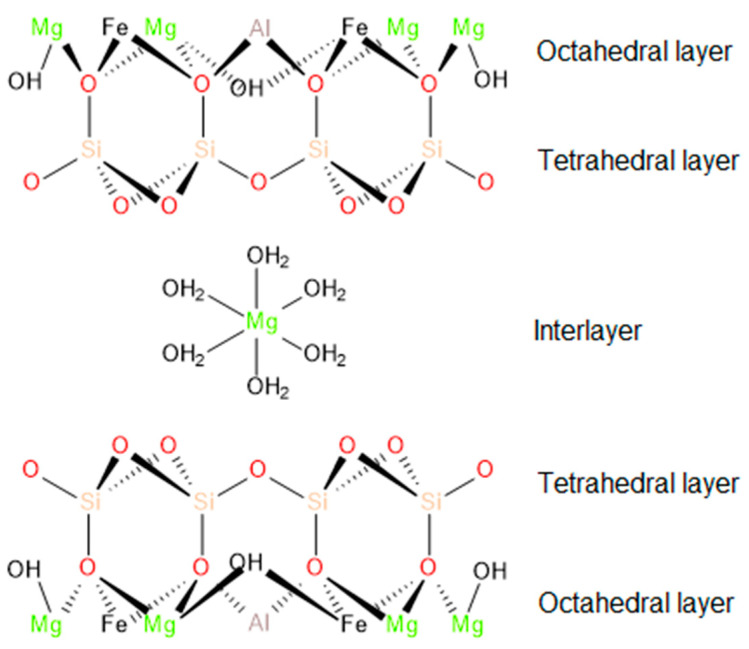
Structure of vermiculite.

Thermally expanded vermiculite (TE-VMT) with a particle size up to 1.6 mm was provided by Perlit Polska (Puńców, Poland). The annealing process was carried out at a temperature of 1260 °C, and the chemical composition according to the manufacturer’s data is 38.0–49.0% SiO_2_, 20–23.5% MgO, 12–17.5% Al_2_O_3_, 0.3–5.4% Fe_2_O_3_, 5.2–7.9% K_2_O, 0–1.2% FeO, 0.7–1.5% CaO, 0–0.8% Na_2_O, 0–1.5% TiO_2_, 0–0.5 Cr_2_O_3_, 0.1–0.3% MnO, 0–0.6% CI, 0-0.6% CO_2_, 0–0.2% S. The exemplary structure of vermiculite based on the literature reports [30,47] is presented in Figure 1. The vermiculite filler is marked as W in the further parts of the study.

**Figure 2:**
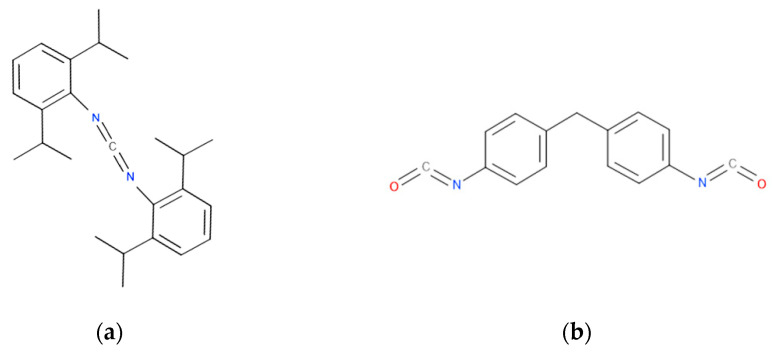
Chemical formula of chain extenders (CE): bis(2,6-diisopropylphenyl)carbodiimide (KI) (**a**) and 4,4′-Methylenebis(phenyl isocyanate) (MDI) (**b**).

Two different chain extenders (CE) have been used for the modification of PLA–TEVMT composites. The first one is BioAdimide^®^ 100 Powder (Lanxess, Koln, Germany), referred to in a further part of the manuscript as KI. This modifier is based on a bis(2,6-diisopropylphenyl)carbodiimide with a linear formula C_25_H_34_N_2_, with minimum carbodiimide content of 10.5%, density of 0.97 g/cm^3^, and a melting temperature of about 50 °C. As a second chain extender reactive with both the filler and the polymer, 4,4′-methylenebis(phenyl isocyanate) (MDI) was used, delivered by Sigma Aldrich (Germany), with a linear formula of CH_2_(C_6_H_4_NCO)_2_, and density of 1.18 g/cm^3^ (25 °C). The chemical formulas of both CEs are presented in Figure 2.

The fixed amounts of the filler (500 g) were placed in a laboratory vacuum dryer and dried for 12 h at a temperature of 75 °C in order to remove the moisture from the material. Next, 2 wt%, according to the mass of the inorganic filler, of the KI and MDI, respectively, were physically mixed with vermiculite using a Retsch GM 200 knife mixer for 2 min with a rotational speed of 2000 rpm. After the preliminary mixing, the compositions of filler with CE and the unmodified filler were introduced to a ball mill with a ball-to-substrates mass ratio of 10:1. The milling lasted 120 min, and the samples were again subjected to an oven drying process, also at a temperature of 75 °C. After that, the samples were sieved using 50 µm sieve by Fritsch Analysette 3 siever.

Constant amounts (20 wt%) of each filler, labeled W, W-KI, and W-MDI, respectively, were physically mixed with the PLA granulate and dried under vacuum at 60 °C for 12 h. Next, all the materials were mixed in a molten state using a ZAMAK EH-16.2D twin-screw co-rotating extruder. The processing was realized at the maximum temperature of 190 °C and rotational speed of the screws of 100 rpm. The extrudates were pelletized after cooling in forced airflow. Specimens with dimensions according to ISO 527 were formed using a Battenfeld PLUS 35 hydraulic injection molding machine operating at the maximum processing temperature of 210 °C. The injection molding process was conducted under the following conditions: mold temperature of 50 °C, injection speed of 75 mm/s, forming pressure of 72 MPa, and cooling time of 50 s.

### 2.2. Methods

The characterization of the particle size distribution of vermiculite subjected to ball milling with and without the modifiers was conducted using a laser particle sizer Fritsch ANALYSETTE 22 apparatus operating in the range of 0.08–2000 μm.

A scanning electron microscope (SEM), model Vega 5135MM produced by the Tescan (Brno, Czech Republic), was used to assess the structure of composites. The structures of the gold-sputtered cryofractured surfaces of the injection-molded samples were assessed with an accelerating voltage of 12 kV.

The Fourier transform infrared spectroscopy (FTIR) measurements were realized using a spectrometer Jasco FT/IR-4600, at room temperature (23 °C) in a mode of Attenuated Total Reflectance (ATR-FTIR). A total of 64 scans at a resolution of 4 cm^−1^ was used in all cases to record the spectra.

The specific weight of applied filler and resulting composites was determined using an Ultrapyc 5000 Foam gas pycnometer from Anton Paar (Austria). The following measurement settings were applied: gas, helium; target pressure, 10.0 psi (for filler) and 18.0 psi (for composites); flow direction, sample first; temperature control, on; target temperature, 20.0 °C; flow mode, fine powder (for filler) and monolith (for composites); cell size, small, 10 cm^3^; preparation mode, pulse, five pulses (for filler) and flow, 0.5 min (for composites); number of runs, 5.

The theoretical values of composites’ density and, based on them, an evaluation of porosity, were calculated according to the simple rule of mixture, expressed by the following:

ρ_theo_ = ρ_m_ × (1 − φ) + ρ_f_ × φ (1)

where: ρ_theo_ is the theoretical density of the composite, g/cm^3^; ρ_m_ is the density of the matrix, g/cm^3^; ρ_f_ is the density of the filler, and g/cm^3^; φ is a volume fraction of the filler.

Using obtained values of the density, the composite’s porosity p was calculated:

p = ((ρ_theo_ − ρ_exp_)/ρ_theo_) × 100% (2)

where p is the porosity of the material, %; ρ_theo_ is the theoretical value of density, g/cm^3^; and ρ_exp_ is an experimental value of the density of the composite, g/cm^3^.

DSC measurements were performed using a Netzsch DSC 204 F1 Phoenix^®^ apparatus with aluminum crucibles and approximately 5 mg samples under nitrogen flow. All the samples were heated up to 210 °C and held in the molten state for 10 min, which was followed by cooling down to 20 °C. Heating and cooling rates were equal to 10 °C/min. This procedure was conducted twice to evaluate the DSC curves from both the first and the second melting procedures and gain broad information about pure PLA and the composites’ thermal properties. The crystallinity level was calculated with the following formula:

(3) $$X_{C}=\frac{\Delta H_{M}-\Delta H_{CC}}{\left(1-\varphi \right)\cdot \Delta H_{MPLA}}\cdot 100\%$$

where Δ*H_M_* is the melting enthalpy, Δ*H_CC_* is cold crystallization enthalpy, Δ*H_MPL_* is melting enthalpy corresponding to a 100% crystalline PLA 93.6 J/g [48], and φ is the filler amount.

Heat deflection temperature (HDT) was determined with a CEAST HV3 apparatus. Measurements were performed in an oil bath following the ISO 75 standard. The HDT B type experiment was prepared with a heating rate of 120 °C/h and a load of 0.45 MPa.

The dynamic mechanical properties of the samples measuring 10 × 4 × 50 mm were studied using the DMTA method in a torsion mode, operating at 1 Hz frequency in a temperature range between 25 °C and 110 °C, and at a heating rate of 2 °C/min. The analysis of the effectiveness of the filler is based on the determination of the “*C*” factor according to the following formula [49]:

(4) $$C=\frac{{\left({G^{\prime }}_{g}/{G^{\prime }}_{r}\right)}_{composite}}{{\left({G^{\prime }}_{g}/{G^{\prime }}_{r}\right)}_{matrix}}$$

where *G_’g_* and *G’_r_* are, respectively, the values of storage modulus determined in the glassy state and the rubbery state after passing the material’s glass transition.

The following equation describes the reinforcement efficiency (r) according to Einstein [50]:

(5) $$r=\frac{\left(\frac{G\prime_{c}}{G\prime_{m}}\right)-1}{V_{f}}$$

where *G’_c_* and *G’_m_* are the storage modulus of composite and unmodified polymeric samples, referred to as *V_f_*, which is the volume fraction of the filler.

The adhesion factor (*A*) of the composite samples was calculated according to the formula proposed by Kubát, Rigdahl, and Welander [51]:

(6) $$A=\frac{1}{1-V_{f}}\frac{\tan\delta_{c}}{\tan\delta_{m}}-1$$

where tan*δ_c_* and tan*δ_m_* are damping factor values of the composite and the matrix, respectively, and *V_f_* is the volume fraction of the filler.

From the results of dynamic mechanical analysis, the volume fraction of macromolecular chains constrained by the filler particles was calculated, based upon the information presented by various researchers [52], using the following:

(7) $$C_{vol}=1-\left(\frac{\left(1-C_{0}\right)W}{W_{0}}\right)$$

where *C_vol_* is the volume fraction of the immobilized polymer chains, %; *C*_0_ is the volume fraction of the immobilized chains in pure PLA (taken to be 0), %; and *W* and *W*_0_ are the energy loss fractions for an analyzed sample and pure PLA, respectively. Energy loss fraction W can be calculated from the tan *δ* by the following:

(8) $$W=\frac{\pi \cdot tan\delta }{\pi \cdot tan\delta +1}$$

The mechanical properties of pure PLA and PLA composites were examined in the static tensile test according to the European Standard PN-EN ISO-527 using a Zwick RoellZ010 universal testing machine with a 10 kN nominal force. The tests were performed with 1 mm/min crosshead speed during Young’s modulus evaluation and 10 mm/min in the remaining range.

The impact strengths of the 10 × 4 × 15 mm unnotched samples were measured by the Dynstat method (DIN 53435).

The hardness evaluation was carried out using a durometer HBD 100-0 Shore D from Sauter GmbH according to the ISO 868 standard.

Flammability tests of PLA samples using a microcalorimeter pyrolysis combustion flow PCFC (Pyrolysis combustion flow calorimeter) were carried out according to the ASTM D7309-2007 standard. The microcalorimeter is a device designed to determine the rate of heat release by materials. The test method is based on the measurement of heat and oxygen loss during thermal decomposition of a small sample of the material. The decomposition is carried out under an inert gas atmosphere in the temperature range 150–750 °C with a heating rate of 1 °C/s. Next, pyrolysis gases are oxidized in a high-temperature furnace at 900 °C for complete oxidation.

## 3. Results and Discussion

### 3.1. Characterization of the Filler

**Figure 3:**
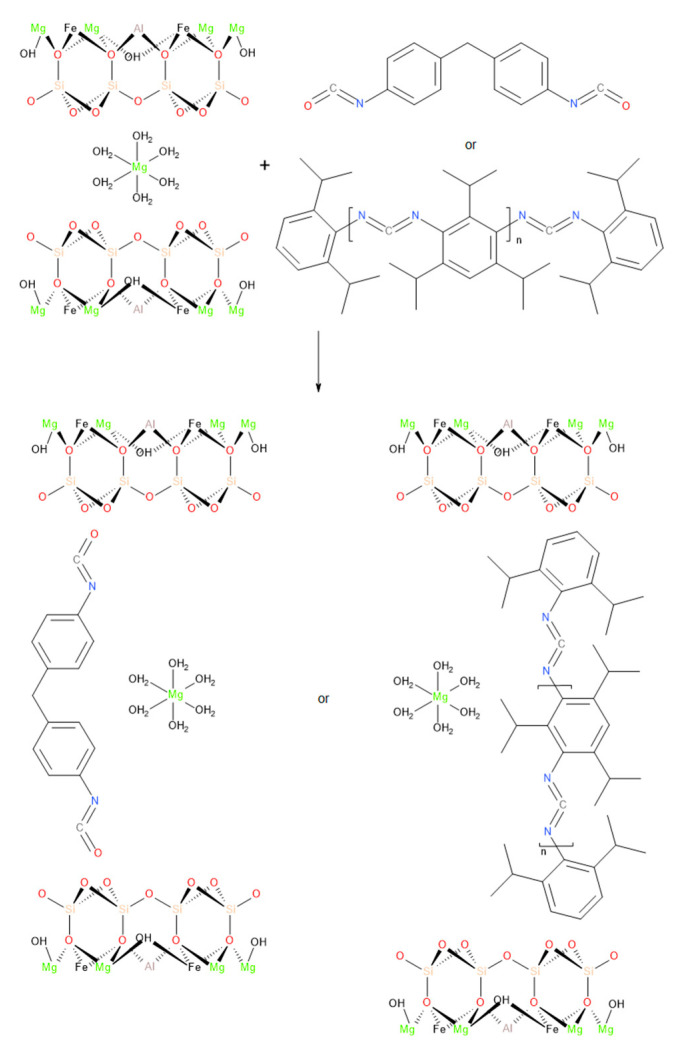
Proposed scheme of interactions between vermiculite and CE.

**Figure 4:**
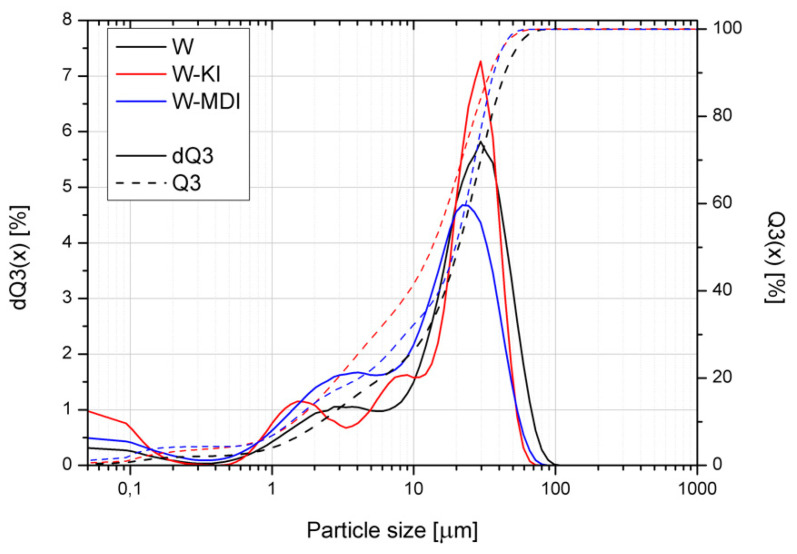
The particle size distribution of vermiculite before and after mechanochemical modification.

Applied modifications of vermiculite resulted in changes in the filler’s structure. One of the parameters changed after modification was the density of vermiculite. The vermiculite subjected to dry ball milling was characterized by a density equal to 2.6337 g/cm^3^, while modifications with KI and MDI reduced the density to 2.5160 and 2.6279 g/cm^3^, respectively. Density decrease is associated with the intercalation of vermiculite, caused by introducing applied compounds into the interlayer, presented in Figure 3. Similar effects were noted in other papers [53]. As a result, the interlayer distance was increased, and the free volume was enhanced. The KI modifier caused a more substantial impact than MDI, which was attributed to the differences in particles’ size (see Figure 4).

**Figure 5:**
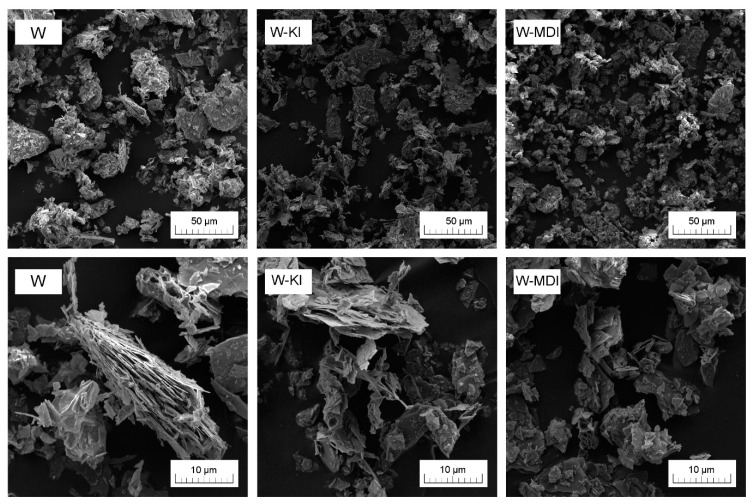
SEM images of the fillers taken with using magnification of 1000× and 5000×.

Figure 4 shows the filler particle size analysis results in the form of the cumulative size distribution Q3 (x) and histogram dQ3 (x) plots as a function of particle size. It can be seen that the use of ball milling with the simultaneous introduction of chain extenders resulted in an increased share of smaller filler fractions, with the size distribution significantly expanded, while the observed tendency to agglomerate formation was absent. This is confirmed by the SEM images shown in Figure 5. The largest share of the smallest filler particles was recorded for W-KI, which may result from a change in the process conditions (i.e., the wet process’s implementation [54]), which results from the low KI melting point. Considering the increased fraction of the smallest particles, in the range below 0.1 µm, it can be assumed that the addition of the filler caused organofunctionalization of the expanded vermiculite and an additional intercalation of its structure. In the case of the isocyanate-modified filler (W-MDI), this effect is much smaller, which may be due to the much greater reactivity of the NCO– groups with hydroxyl groups, which probably caused only the surface modification of the filler and not a change in the nature of the process conditions. SEM images taken with two different magnifications presented in Figure 5 are consistent and confirm the particle size analysis results. The shape of ground vermiculite showed a layered morphology, which is typical for this mineral [55]. In the W-KI composite case, the most significant fragmentation of the filler structure in the grinding process was noted. For both modified variants of the filler, a significant reduction in the number of large vermiculite fractions was noted. In addition, for W-KI/MDI, a significant reduction in expanded particles and the breakdown of the packages into individual plates is observed.

**Figure 6:**
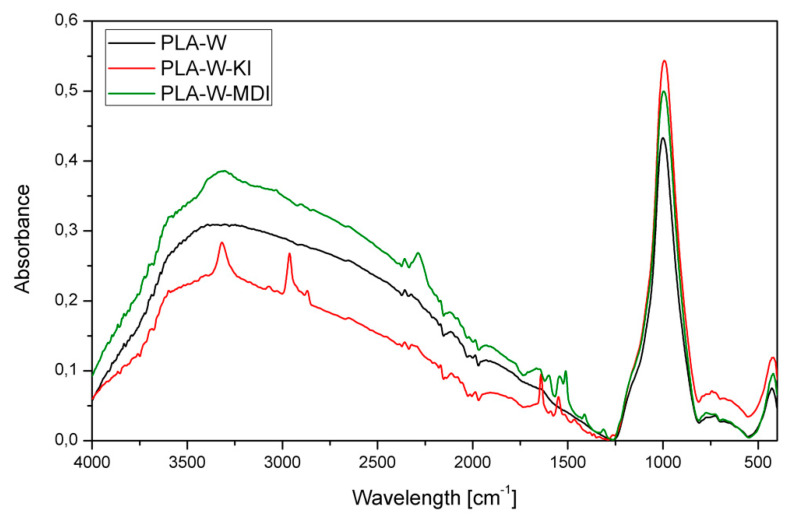
FTIR spectra of unmodified vermiculite and series subjected to mechanochemical modification.

Figure 6 presents the FTIR spectra of unmodified and modified with MDI and KI vermiculite after ball milling. The spectra related to pure expanded vermiculite shows characteristic bands starting from the one at 3670 cm^−1^ related to the valence vibration of the octahedral hydroxyl Mg_2_AlOH. Further, there are less visible absorption bands at 1638 cm^−1^ corresponding to the vibrations of the H–O–H groups. The band located at 1071 cm^−1^, which is also the most intense one, comes from the stretching vibrations associated with the Si–O bonds of amorphous vermiculite silica. The weak peak at approximately 746 cm^−1^ corresponds to the vibration in the deformation plane of the Al–O–Si bonds of vermiculite. The last band that appeared in the considered wavelength range is located at 450 cm^−1^ and includes bending vibrations of the Si–O–Si bond. Both modified series of the filler show additional bands originated from the used modifier (MDI or KI). The W-MDI curve shows the characteristic diisocyanate peak at 2260 cm^−1^. It is related to the presence of N=C=O– stretching groups in the MDI molecule. The intensification of the flattened peak at 3370 cm^−1^ is due to the presence of the amino group N–H stretching bonds. In addition, an increase in absorbance at 1600 cm^−1^ can be seen from the C = O stretching groups. The increase in the bands’ intensification compared to vermiculite can be observed at 1071 and 450 cm^−1^. In the W-KI sample, apart from the characteristic absorption bands coming from the filler, characteristic absorption bands from the used chain extender were observed. The band at a wavelength of about 3000–2800 cm^−1^ is related to the CH_2_ and CH_3_ groups’ presence. The peaks observed at a wavelength of about 1600–1500 cm^−1^ are characterized by –NH_2_ (1620 cm^−1^) and NH (1520 cm^−1^) groups. The presence of the C–N moieties is confirmed by the peaks at wavelengths of 1460–1350 cm^−1^ and 1250 cm^−1^. According to Oliviera et al., the presence of additional bands in 2962 and 2857 cm^−1^ for W-KI may be attributed to the proper realization of the carbodiimide surfactant absorption within the vermiculite structure [56].

### 3.2. Influence of Filler Addition and Its Surface Treatment on PLA-Composites’ Properties

**Figure 7:**
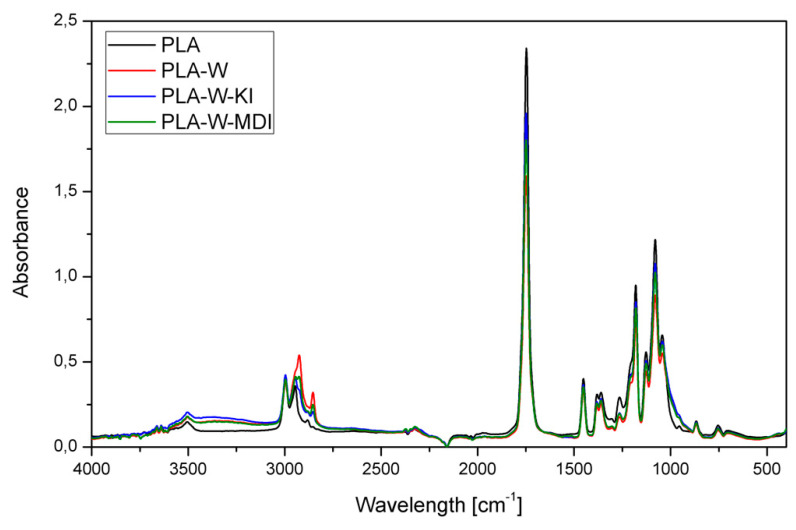
FTIR spectra of polylactide (PLA) and PLA composites filled with vermiculite subjected to various chemical treatments.

Figure 7 shows the FTIR spectra of unmodified PLA and PLA filled with three types of vermiculite. All spectra present the absorption bands typical for PLA resulting from its chemical structure: asymmetrical (2997 cm^−1^) and symmetrical (2946 cm^−1^) stretching (2877 cm^−1^) of –CH–, –C=O carbonyl stretching (1748 cm^−1^), –CH_3_ bending (1456 cm^−1^), –C–H– deformation including symmetric and asymmetric bending (1382 and 1360 cm^−1^), −C=O bending (1263 cm^−1^), –C–O– stretching (1181, 1127 and 1079 cm^−1^), –OH bending (1047 cm^−1^), –CH_3_ rocking (956 cm^−1^), and –C–C– stretching (867 cm^−1^) [57,58,59,60,61]. In the majority of the absorption bands, a decrease in their intensity was noted in the case of composite materials compared to the unmodified PLA. An additional absorption band was recorded for all three composite samples at wavelengths of 2924 and 2853 cm^−1^, which belong to aliphatic C–H stretching attributed to organophilization of vermiculite, according to Gomes et al. [62]. The highest intensity of the first one was recorded for the PLA-W sample, while it was visible only as an inflection for PLA-W-KI. In the composite filled with the chemically unmodified vermiculite, the absorption band at 2853 cm^−1^ was partially limited when the surface modification was applied.

**Figure 8:**
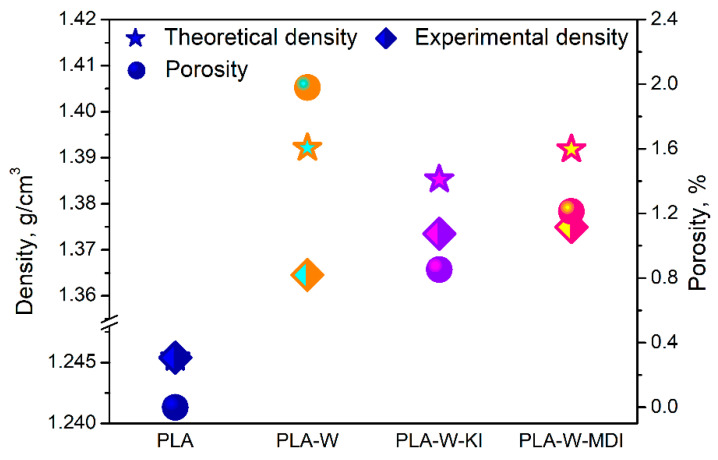
The values of density and porosity of PLA and its composites.

Figure 8 presents the values of density and porosity of the unfilled PLA and the prepared composites. Theoretical values were calculated based on the density of the unfilled PLA and the applied fillers, as mentioned above. It can be seen that there are differences between analyzed composites due to the structural changes caused by the filler modification. Both modifications of vermiculite increased the measured values of composites’ density. Such an effect could be attributed to the increased interlayer distance in the filler, so that PLA macromolecules could insert the interlayer, and the free volume could be reduced. Therefore, modification of vermiculite resulted in the drop of porosity. The effect was stronger for the KI modifier because of the more significant increase in interlayer distance of vermiculite. All determined values of the porosity are rather small and should not be related to mechanical properties changes.

**Figure 9:**
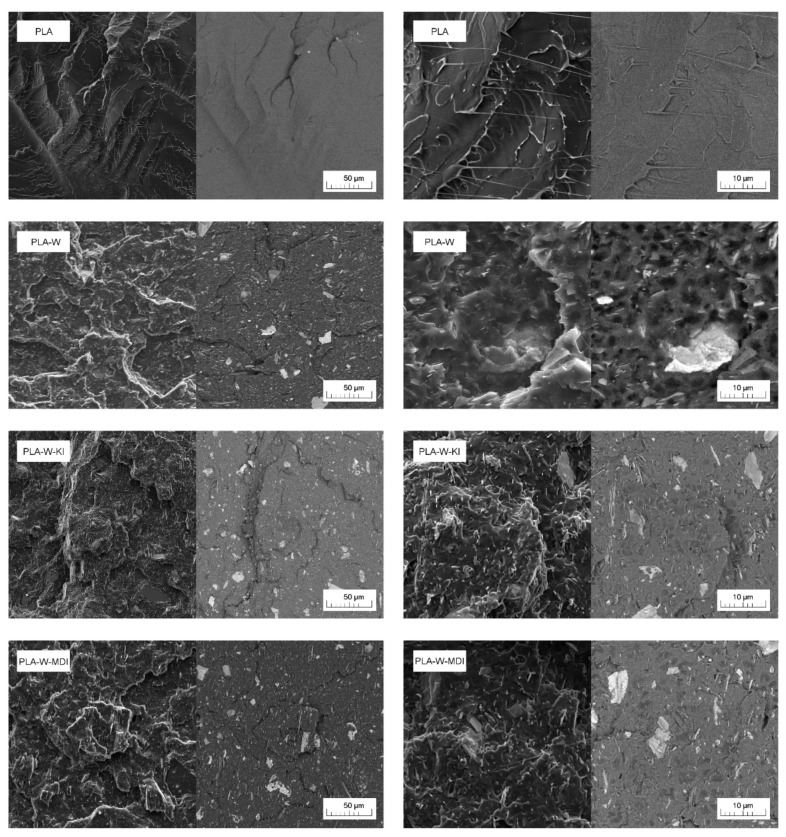
SEM images of PLA and PLA composites taken in SE and BSE mode with using 1000× and 5000× magnification.

Figure 9 shows the SEM images of brittle fractures of PLA and its composites made in the secondary electrons (SE, left image) and back-scattered electrons (BSE, right image) techniques to present the exact location of the filler in the polymer matrix. Additionally, for each material sample, images were taken at two different magnifications. Unmodified PLA showed a typical amorphous polymer structure with large brittle cracks [63]. For all three composites, good filler dispersion in the PLA matrix was noted. In the case of composites containing the vermiculite modified with carbodiimide, a significant increase in the presence of single expanded filler plates was noted. In the case of the sample containing an isocyanate pre-treated filler, larger particles were less common than for the PLA-W composite. This observation is consistent with the previously discussed particle size analysis and the morphology of ball milling fillers. According to the literature [29], organofunctionalized vermiculites (OVMT) are characterized by higher compatibility with the PLA matrix, which results in a more favorable dispersion of fillers into the matrix. The more extensive fracture surface of composites compared to unmodified PLA may result from both the increased degree of crystallinity caused by the nucleating ability of the filler, as well as the presence of the effect of more difficult crack propagation caused by the presence of dispersed clay resulting in a large number of stress concentration points and, in effect, higher toughness [29,63]. Good adhesion at the polymer–filler interface was noted for both the composite produced using the unmodified inflator and those preprocessed with the presence of CE.

**Figure 10:**
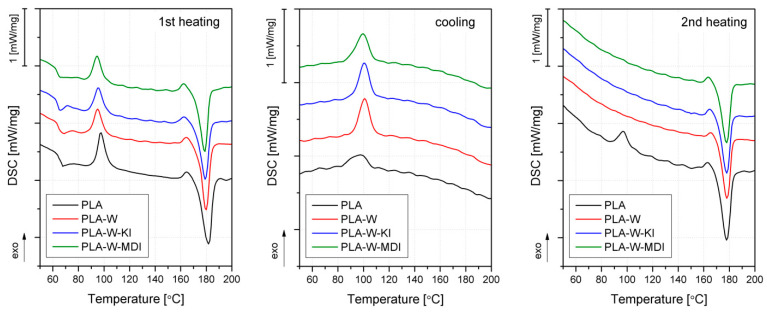
DSC heating and cooling curves of PLA and PLA-W composites.

The DSC curves obtained during the first heating, cooling, and second heating of PLA and its composites are presented in Figure 10. The values of glass transition, melting, crystallization, and cold crystallization temperatures (Tg, Tm, Tc, and Tcc, respectively), as well as the crystallinity degree Xc, are collected in Table 1. During the first heating, all the studied samples undergo several processes, starting with a glass transition of about 65 °C. An exothermic peak around 95 °C can be attributed to the so-called cold crystallization, which takes place when a polymer is quenched during cooling from melt and does not have enough time to develop a crystalline structure. Finally, the melting of PLA and the vermiculite-filled composites is indicated by a large endothermic peak around 180 °C. This behavior is typical for polylactide-based materials, characterized by a low crystallization rate [64]. The shape of the curve is almost identical for the vermiculite-filled samples and the unmodified PLA. The most visible difference is a small shift of cold crystallization peak towards lower temperatures recorded for the composite samples. As shown in Table 1, the Tcc of the vermiculite-filled materials is about 2 °C lower than the one of PLA. Even though this difference is small, it can indicate the nucleating effect of the filler on the matrix polymer. Fernandez and Fernandez described similar results in their work on polylactide/organovermiculite nanocomposites [65]. The influence of the filler on the crystallization of PLA is even more noticeable on the DSC thermogram recorded during cooling. All the studied materials show an exothermic crystallization peak; however, it is much more developed in composite materials. During the subsequent heating, the vermiculite-filled materials do not undergo cold crystallization, which means that cooling at a rate of 10 °C/min is sufficient to allow for full development of a crystalline structure. The nucleating effect of the mineral filler is also indicated by a substantial increase in the crystallinity degree. The Xc value calculated during the second heating for PLA is 49.3%, and in the case of the composites, it is in the range of 54.8–61.1%. Interestingly, the increase of crystallinity is not accompanied by changes in the melting temperature. The Tm values recorded during the second heating are almost the same, which indicates that the addition of the filler does not change the size or order of perfection of the spherulites [29]. The influence of the filler on the crystallization of PLA can be also altered by its chemical modification. Application of the isocyanate-modified vermiculite resulted in the highest Xc value accompanied by lowered Tcc, whereas the PLA-W-KI sample showed even lower crystallinity than the composite containing the unmodified filler. The lower nucleation ability of the carbodiimide-modified vermiculite can indicate the filler’s good affinity—its particles serve as constraints, limiting the mobility of PLA macromolecules, and do not allow for creating a highly ordered structure [41]. This can also result from the more intercalated structure of W-KI: some of the polymeric chains can enter the gap between the vermiculite platelets, but they cannot form a crystalline structure in those confined spaces. In the case of the W-MDI-filled composites, the distance between the vermiculite layers is smaller, a smaller number of the macromolecules is able to penetrate the gaps and most of them arrange freely outside of the filler particles, which allows for a more ordered crystalline structure. The glass transition determined during the first heating was not influenced by the addition of the unmodified vermiculite, while incorporating the modified fillers caused a comparable decrease in about 3 °C of this parameter.

**Figure 11:**
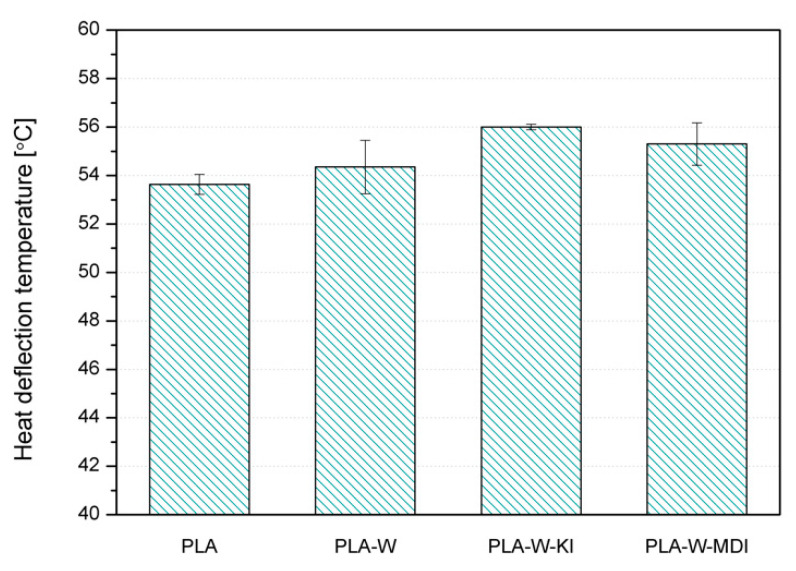
Heat deflection temperature values of PLA and PLA-W composites.

The effect of the addition of an inorganic filler and modification of its surface on the thermomechanical properties of composites with a PLA matrix was assessed under static and dynamic loading conditions. Measurements of thermomechanical stability under static conditions were carried out using the heat deflection temperature (HDT) method, while the analysis of thermomechanical properties in the field of non-destructive dynamic loads was carried out using DMTA. The list of mean HDT values obtained for PLA and its composites is presented in Figure 11. According to Wu et al., the possibility of improving HDT due to the partial crosslinking of the PLA structure as an effect of interactions with CE bonded to filler may be neglected [66]. An increase in HDT value in PLA is usually associated with an increased share of the crystalline phase [67]. Moreover, the effectiveness of fillers’ influence on the increase of HDT is also related to its influence on the polymer’s crystalline structure [6]. In the considered case, vermiculite shows nucleating ability for PLA, however the observed changes in the degree of crystallinity are mainly due to the low mold temperature during the injection molding of the specimens. Despite the lack of substantial changes in the degree of crystallinity of composites, a slight increase in HDT of composite samples can be observed. The influence of the surface modification is also visible. The composites manufactured using W-KI and W-MDI revealed the highest thermomechanical stability.

**Figure 12:**
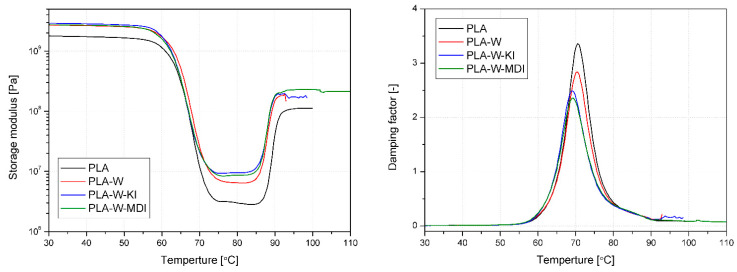
DMTA storage modulus and damping factor vs. temperature plots of PLA and PLA-W composites.

The results of the evaluation of thermomechanical properties under dynamic load conditions (DMTA) are presented in the form of graphs of changes in storage modulus (G’) and damping factor (tanδ) vs. temperature in Figure 12. Additional information about glass transition temperature (Tg) determined as a peak of tanδ curve and thermomechanical parameters calculated according to Equations (4–7) are collectively presented in Table 2. Comparing the changes in G’ of PLA samples and the composites, an apparent increase in stiffness in the entire considered temperature range can be noticed. While in the glassy state, composites are characterized by similar G’ values, after exceeding the Tg, one can observe the increase in the difference between the materials. The highest G’ values in the rubbery state were recorded for PLA-W-KI. However, both materials containing modified vermiculite were characterized by improved thermomechanical stability in comparison with PLA-W. Considering the changes in the degree of crystallinity assessed by DSC and the structure of composites assessed by the SEM method, it can be concluded that while the improvement in PLA-W-MDI stiffness results from the increased degree of crystallinity, in the case of PLA-W-KI, the dominant effect must be an increase in the degree of filler dispersion. The samples with the carbodiimide-modified filler have a degree of crystallinity comparable to the PLA-W series.

The values of glass transition obtained from DSC and DMA vary, which is the common apparatus effect [68,69]; however, the tendency in the changes of these parameters are the same. For both composites containing the modified fillers, the Tg is lowered. Taking into account the results presented by Laske et al. [46], who linked the decrease in Tg to crosslinking of PLA, it can be concluded that the effect is similar for the CE-functionalized fillers. While the use of KI and MDI as chain extenders is associated with the introduction of compounds having two functional groups, introducing a modified filler containing many functional groups on its surface may cause the appearance of active centers for the formation of 3D spatial PLA structures. At the same time, it should be noted that the difference between the results for the obtained DMA data is smaller. In the case of DSC analysis, the results of the glass transition are not affected by adhesive phenomena at the interface, but only information is obtained about the modification of the structure of the polymer matrix, which may result from a change in the degree of crystallinity or molecular weight of the polymer [70]. The data obtained by the DMA method is much more comprehensive, and the result is influenced by both changes in the polymer matrix and changes in interfacial adhesion. Referring to the previously published results obtained for PLA composites modified with OVMT, in which the increase in Tg was univocally related to the improvement of interfacial adhesion, in the case in question, it can be considered as co-occurring with changes in the molecular weight of the polymer. It can be assumed that the reduced difference between the Tg determined by the DMA method for PLA, PLA-W, and PLA-W-KI/MDI results from proper interfacial adhesion.

Thermomechanical parameters calculated from storage modulus values of composites in reference to unmodified PLA (i.e., *C* factor and *r*) show a similar tendency. In the case of *C* factor, lower values suggest greater effectiveness of the filler on thermomechanical stability changes [49]. The changes in the reinforcing efficiency are analyzed differently because the increase in the value of *r* is related to the greater propensity rather than the reinforcement of the structure by filler. The effectiveness of interaction with the polymer matrix described by both parameters is more significant for the composites with the modified fillers than for the PLA-W composite, and the best effect was observed for W-KI particles’ interaction. The lower the adhesion factor, the more distinct interfacial interactions between the polymer and a filler [71]. The use of both types of modifications resulted in reducing Af, especially for the PLA-W-KI composite. The values of the C_vol_ are relatively low compared to the volume fractions of applied fillers. Nevertheless, such an effect can be attributed to particles’ aggregation and disproportion between filler content and interfacial area. Therefore, the filler particles are not able to fully interact with polymer macromolecules. Such an effect was also observed by Bindu and Thomas [72], even for 3 phr content of ZnO nanoparticles. Nevertheless, the applied vermiculite modifications significantly enhanced the volume fraction of constrained polymer chains, indicating the enhanced interfacial interactions.

**Figure 13:**
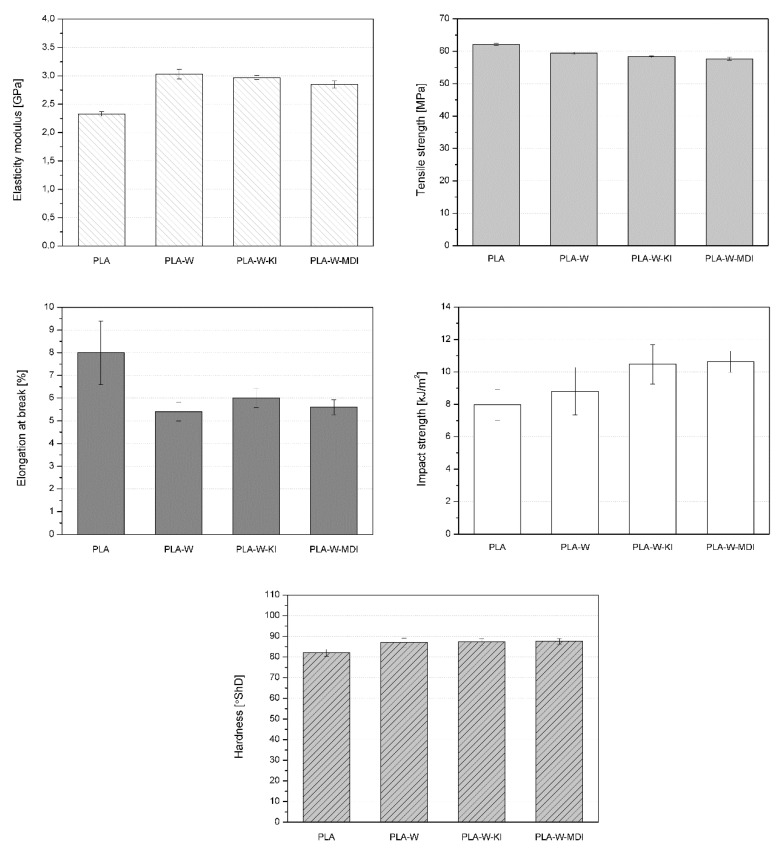
Mechanical properties of PLA and PLA-W composites.

The mechanical properties such as elasticity modulus, tensile strength, and elongation at break determined during the tensile test, impact strength, and hardness are shown in Figure 13. Polylactide is a stiff and robust material, characterized by a tensile modulus of 2.25 GPa, a tensile strength of 61 MPa, and elongation at a break of 8%. The addition of 20 wt% of vermiculite, regardless of its type, increases Young’s modulus, which is a commonly observed effect [73]. As the applied filler belongs to the group of soft minerals (Mohs hardness of 2–3 [74]), the stiffening effect is presumably due to the interactions of the phases in the composite, not the presence of rigid filler particles. The filler content is high enough to create an internal structure of the mineral particles, which act as a reinforcement and cause an increase of the composite’s tensile modulus [75]. The increase of the composites’ stiffness can also be explained by their high crystallinity [76]. However, the sample characterized by the highest Xc value also presents the lowest Young’s modulus among the vermiculite-filled materials. Therefore, it can be concluded that high crystallinity and lowered stiffness of the isocyanate-modified composites result from a common reason: the applied modifying agent facilitates macromolecules’ movements during the creation of the crystalline phase and also during the tensile test. Nevertheless, it needs to be noticed that the differences of the tensile modulus between the three studied composites are small and should not influence their application potential.

The tensile strength of the polymeric materials also decreases due to the addition of vermiculite. This behavior presumably results from the presence of filler aggregates, which act as points of stress concentration. What is more, when the filler particles meet each other, the crack can propagate more easily, facilitating the breaking of the sample [77]. Nevertheless, consider that even in the composite filled with 20 wt% of unmodified silicate, the tensile strength is only 3 MPa lower compared to PL; this result is highly satisfying. Surprisingly, the filler modification did not result in an increase in tensile strength. This phenomenon can be explained by the fact that the tensile strength is more dependent on the presence of large filler aggregates and structure imperfections rather than the matrix–filler interactions. Similar behavior was noted in the elongation at break, which decreases due to the addition of the filler. It can also be connected to the more complicated structure of the multiphase materials, where the filler aggregates, porosities, or interface imperfections result in an easier fracture of the sample. Interestingly, the impact strength evaluation shows an entirely different result. As can be seen in Figure 13, the vermiculite-filled composite is more resistant to impact than the unfilled polymer, and the best results are achieved by the PLA-W-KI and PLA-W-MDI samples. This behavior indicates the presence of strong filler­–filler and polymer–filler interactions, which need more energy to be destroyed during the impact. The composite samples have a higher degree of crystallinity, which also results in better impact strength. In the samples filled with unmodified vermiculite, there is a stable structure of contacting filler particles, which needs to be destroyed along with the polymeric matrix during the impact. In the composites containing the modified filler, there are also strong interactions between the phases, which also boost the impact strength. Therefore, different phenomena such as the increased crystallinity, filler distribution in the sample, and the polymer’s adhesion to the vermiculite particles result in a substantial increase of impact strength. This outcome is especially advantageous, as it limits one of the PLA’s main drawbacks, which is brittleness.

Unlike the impact strength, hardness is barely affected by the addition of the filler to the polymer. A slight increase can be observed, and all the composite types show almost the same hardness value. This result presumably affects the samples’ increased crystallinity degree because vermiculite, as a soft mineral [74], is unlikely to provide higher hardness values.

**Figure 14:**
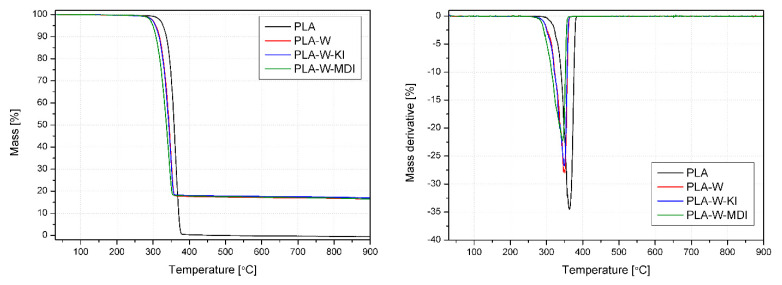
TG and dTG curves of PLA and PLA-W composites.

The mass loss curves registered during the thermogravimetric analysis and its first derivative (dTG) are presented in Figure 14 and Table 3. The thermal decomposition of PLA in nitrogen is a single-step process that takes place in the range of 300–400 °C. The beginning of the thermal degradation was understood as the temperature of 5% mass loss at 327.2 °C, and its maximum rate was determined as the dTG curve peak at 364.3 °C, which is typical for this polymer [78]. The whole sample mass was degraded and volatilized during the experiment, as the residual mass is 0%. The vermiculite-filled composites begin the thermal decomposition at approximately 20–30 °C lower temperatures than the unmodified PLA. Similar behavior was also described by Fernandez et al. in the case of organically modified vermiculite-filled polylactide [29]. It was explained by a lower molecular weight of the composite samples, which resulted from thermo-mechanical degradation of the polymers during the melt processing. As chain extenders were used to modify the composites, the decrease of molecular weight is unlikely, so this behavior may be an effect of the filler dehydration, which takes place below 200 °C [29], and the volatilization of the modifying agents from the W-KI W-MDI fillers. This hypothesis is supported by the fact that the addition of the mineral filler mostly affects the beginning of the decomposition (there is a larger difference between the T_5%_ values of the composites and pure PLA in comparison with dTG_T_), and there is a visible difference between the samples filled with isocyanate- and carbodiimide-modified vermiculite. What is more, the composite samples’ residual mass is approximately 17%, which is lower than their filler content. Considering that the whole PLA mass is burned during the test, it needs to be assumed that about 15% of the filler is also degraded in the studied process. However, it needs to be stressed that even for the PLA-W-MDI samples, which are characterized by the lowest thermal stability among the researched materials, the decomposition begins at a temperature 100 °C higher than the standard processing temperature. Therefore, addition of the modified vermiculite should not limit the processing window of PLA-based composites.

**Figure 15:**
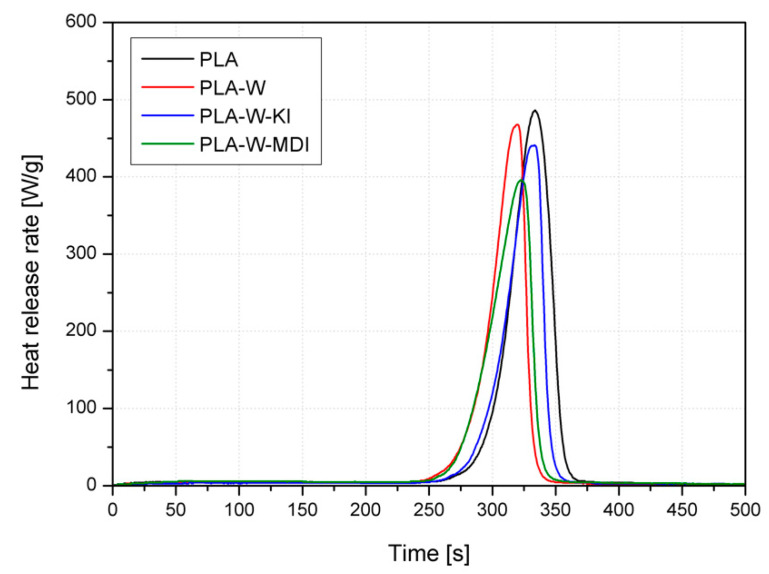
Results of PCFC of PLA and PLA composites.

Table 4 and Figure 15 collectively present the results of microcalorimetric investigations of the samples made of unmodified PLA and PLA-composites containing 20 wt% of the inorganic filler subjected to different surface modifications. All the curves of the analyzed materials had a single-stage course, and no influence of the applied modifiers on their change was observed. The amount of the filler that could have influenced the run of the curves is presumably not high enough to have an observable effect. However, the applied modifiers affect the values parameters obtained in the combustion process, which clearly shows that they influence this process, and their effects may overlap in the pHRR peak. For the analyzed materials, the lowest values of total heat of combustion (THR), peak heat release rate (pHRR), and heat release capacity (HRC) ratio to the reference material (PLA), were achieved by the material filled with W-MDI. In addition, the appearance of a pHRR peak occurred at the lowest temperature (T_pHRR_) and after the shortest time (t_pHRR_). It should be underlined that HRC, defined as maximum heat release divided by a constant heating rate in a test, is taken as the single best measure for the fire hazard of tested materials [79]. Therefore, the observed changes of the microcalorimetric investigations show the most beneficial fire resistance of the composites containing W-MDI filler. PLA-W composite revealed the highest heat release rate (pHRR) peak values, the total heat of combustion (THR), and heat release capacity (HRC) compared to composites containing modified vermiculite (W-KI and W-MDI). This phenomenon may be related to the discussed structural changes of modified fillers and the composites manufactured with their use. It can be concluded that ball milling with CE’s presence allows the improvement of the intercalation process and provide better dispersion of the filler in a polymeric matrix.

## 4. Conclusions

In the presented study, a novel mechanochemical method was successfully applied to modify the surface chemistry and shape of thermally expanded vermiculite, which was used as a filler in polylactide composites. The conducted tests and experiments proved that both carbodiimide and isocyanate bond with this phyllosilicate and improve its intercalation. It was found that even the unmodified vermiculite is a cost-effective filler for the aliphatic polyester, which improves its crystallinity, thermomechanical stability, and stiffness, but the best results were obtained in the case of the composites with the ball-milled silicate. Both the W-MDI and W-KI fillers presented improved affinity to PLA and good dispersion in the polymeric matrix, but each of them showed a different influence on the property of the composites. Even though modification of vermiculite with carbodiimide resulted in the best interactions of the filler and the polymer, excellent dispersion of its particles, good thermal stability and relatively high elongation at break of the composites, the application of isocyanate caused a notable increase of crystallinity accompanied with elevated impact strength and improved burning resistance. Therefore, it can be concluded that mechanochemical modification of vermiculite is an efficient way to obtain an effective filler tuned for application in different PLA composites.

## Figures and Tables

**Table 1:** Thermal parameters obtained for PLA and PLA-based composites obtained from DSC.

Sample	Tm ^I^	Tcc ^I^	Xc ^I^	Tm ^II^	Tcc ^II^	Xc ^II^	Tc ^c^	Tg ^I^
	[°C]	[°C]	[%]	[°C]	[°C]	[%]	[°C]	[°C]
PLA	181.8	97.5	29.3	177.9	96.8	49.3	97.8	66.5
PLA-W	179.7	95.0	35.2	178.2	-	58.6	100.9	65.8
PLA-W-KI	179.2	95.5	35.8	177.9	-	54.8	100.5	63.4
PLA-W-MDI	178.8	94.5	41.5	177.8	-	61.1	99.5	63.6

^I^—determined during 1st heating, ^II^—determined during 2^nd^ heating, ^c^—determined during cooling.

**Table 2:** Thermomechanical parameters obtained from DMA.

Material	G’30	G’80	Tg	Tanδ at Tg	C	r	A_f_	C_vol_
	[Pa]		[°C]	[-]	-			
PLA	1.78 × 10^7^	2.99 × 10^6^	70.5	3.36	n.a.	n.a.	n.a.	n.a.
PLA-W	2.70 × 10^7^	6.46 × 10^6^	70.5	2.84	0.702	0.058	0.0950	0.0155
PLA-W-KI	2.89 × 10^7^	9.45 × 10^6^	69.0	2.49	0.514	0.108	0.0522	0.0293
PLA-W-MDI	2.75 × 10^7^	8.65 × 10^6^	69.3	2.36	0.534	0.095	0.0827	0.0353

**Table 3:** Thermal parameters obtained for PLA and PLA-based composites obtained from TGA.

Scheme 5.	T_5%_	T_10%_	T_50%_	dTG_T_	dTG_max_	Residual Mass
	[°C]				[%/min]	[%]
PLA	327.2	336.5	357.8	364.3	34.51	0
PLA-W	307.7	336.5	344.4	351.1	28.18	16.49
PLA-W-KI	306.5	317.8	343.1	349.0	26.78	17.01
PLA-W-MDI	299.4	307.7	336.6	344.4	22.23	16.51

**Table 4:** Results of microcalorimetry investigations of PLA and PLA composites.

Sample	pHRR	T_pHRR_	t_pHRR_	HRC	THR
	[W/g]	[°C]	s	J/g·K	kJ/g
PLA	488.6 ± 17.8	383 ± 1	336 ± 5	528.3 ± 22.2	19.8 ± 0.4
PLA-W	455.7 ± 12.7	369 ± 1	322 ± 6	498.0 ± 4.2	16.7 ± 0.1
PLA-W-KI	445.7 ± 7.7	368 ± 2	324 ± 8	483.5 ± 3.5	16.6 ± 0.2
PLA-W-MDI	391.3 ± 6.2	367 ± 2	318 ± 6	423.5 ± 6.4	16.5 ± 0.6
